# Risk factors and molecular epidemiology of intestinal colonization by carbapenem-resistant Gram-negative bacteria in patients with hematological diseases: a multicenter case‒control study

**DOI:** 10.1128/spectrum.04299-23

**Published:** 2024-06-07

**Authors:** Huangdu Hu, Yinping Wang, Jian Sun, Yuting Wang, Junxin Zhou, Qiucheng Shi, Xinhong Han, Yan Jiang, Depei Wu, Xiaojun Huang, Yunsong Yu

**Affiliations:** 1 Department of Infectious Diseases, Sir Run Run Shaw Hospital, Zhejiang University School of Medicine, Hangzhou, Zhejiang, China; 2 Key Laboratory of Microbial Technology and Bioinformatics of Zhejiang Province, Hangzhou, Zhejiang, China; 3 Regional Medical Center for National Institute of Respiratory Diseases, Sir Run Run Shaw Hospital, Zhejiang University School of Medicine, Hangzhou, China; 4 Department of Critical Care Medicine, Lishui Central Hospital, Lishui, China; 5 Department of Clinical Laboratory, Zhejiang Cancer Hospital, Hangzhou, Zhejiang, China; 6 National Clinical Research Center for Hematologic Diseases, Jiangsu Institute of Hematology, The First Affiliated Hospital of Soochow University, Suzhou, China; 7 Peking University People’s Hospital, Peking University Institute of Hematology, National Clinical Research Center for Hematologic Disease, Beijing Key Laboratory of Hematopoietic Stem Cell Transplantation, Beijing, China; University of Mississippi Medical Center, Jackson, Mississippi, USA

**Keywords:** CR-GNB, intestinal colonization, hematological diseases, risk factors, molecular epidemiology

## Abstract

Patients with hematological diseases are considered to be at high risk for intestinal colonization by carbapenem-resistant Gram-negative bacteria (CR-GNB). However, the epidemiological data regarding risk factors and molecular characteristics of intestinal colonized CR-GNB isolates in this population are insufficient in China. A multicenter case‒control study involving 4,641 adult patients with hematological diseases from 92 hospitals across China was conducted. Following culture of collected rectal swabs, mass spectrometry and antimicrobial susceptibility tests were performed to identify GNB species and CR phenotype. Risk factors were assessed through retrospective clinical information. Whole-genome sequencing was used to analyze the molecular characteristics of CR-GNB isolates. This trial is registered with ClinicalTrials.gov as NCT05002582. Our results demonstrated that among 4,641 adult patients, 10.8% had intestinal colonization by CR-GNB. Of these, 8.1% were colonized by carbapenem-resistant *Enterobacterales* (CRE), 2.6% were colonized by carbapenem-resistant *Pseudomonas aeruginosa* (CRPA), and 0.3% were colonized by carbapenem-resistant *Acinetobacter baumannii* (CRAB). The risk factors for CR-GNB colonization include male gender, acute leukemia, hematopoietic stem cell transplantation, β-lactam antibiotic usage, and the presence of non-perianal infections within 1 week. Compared with CRPA-colonized patients, patients using carbapenems were more likely to be colonized with CRE. NDM was the predominant carbapenemase in colonized CRE. This study revealed a high CR-GNB intestinal colonization rate among adult patients with hematological diseases in China, with CRE being the predominant one. Notably, a significant proportion of CRE exhibited metallo-β-lactamase production, indicating a concerning trend. These findings emphasize the importance of active screening for CR-GNB colonization in patients with hematological diseases.

**IMPORTANCE:**

Carbapenem-resistant Gram-negative bacteria (CR-GNB) has emerged as a significant threat to public health. Patients with hematological diseases are at high risk of CR-GNB infections due to their immunosuppressed state. CR-GNB colonization is an independent risk factor for subsequent infection. Understanding the risk factors and molecular characteristics of CR-GNB associated with intestinal colonization in patients with hematological diseases is crucial for empirical treatment, particularly in patients with febrile neutropenia. However, the epidemiology data are still insufficient, and our study aims to determine the intestinal colonization rate of CR-GNB, identify colonization risk factors, and analyze the molecular characteristics of colonized CR-GNB isolates.

## INTRODUCTION

The global dissemination of carbapenem-resistant Gram-negative bacteria (CR-GNB) has emerged as a significant threat to public health (1). In 2017, the World Health Organization (WHO) identified carbapenem-resistant *Enterobacterales* (CRE), carbapenem-resistant *Pseudomonas aeruginosa* (CRPA), and carbapenem-resistant *Acinetobacter baumannii* (CRAB) as important pathogens for which new antibiotics are needed (2). Previous studies have demonstrated that patients infected with CR-GNB have a higher mortality rate than those infected with carbapenem-susceptible strains (3–6).

Patients with hematological diseases are at high risk of CR-GNB infection due to factors such as bone marrow suppression, gastrointestinal mucositis resulting from the use of immunosuppressants and chemotherapy drugs, prolonged hospitalization, neutrophil deficiency, and frequent use of broad-spectrum antibiotics (7). Previous research has identified intestinal colonization by CR-GNB as an independent risk factor for subsequent infection. In cases where the colonized CR-GNB strains breach the intestinal mucosal barrier, severe infections can occur, with mortality rates ranging from 65% to 100% (8, 9). Furthermore, studies have shown that intestinal colonization by drug-resistant bacteria can impact the overall survival and increase mortality rates in patients undergoing hematopoietic stem cell transplantation (HSCT) (10). Consequently, active screening for intestinal CR-GNB colonization in patients with hematological diseases has become a crucial preventive measure for controlling and averting nosocomial infections (11, 12).

The rates of CR-GNB intestinal colonization in patients with hematological diseases vary across different regions worldwide. For instance, an Italian multicenter observational study reported a CR-GNB intestinal colonization rate of 3.8% in patients with hematological malignancies (13). However, currently, there is a lack of epidemiological data on CR-GNB intestinal colonization in patients with hematological diseases in China. This study aims to address this gap by conducting a multicenter observational study; in this study, we determined the rate of CR-GNB intestinal colonization, identified risk factors for colonization, and analyzed the molecular characteristics of colonized CR-GNB isolates in China. The findings from this study contribute to reducing the colonization rate and the incidence of infection and mortality associated with CR-GNB in patients with hematological diseases.

## RESULTS

### Intestinal colonization rate of CR-GNB in patients with hematological diseases

**Fig 1:**
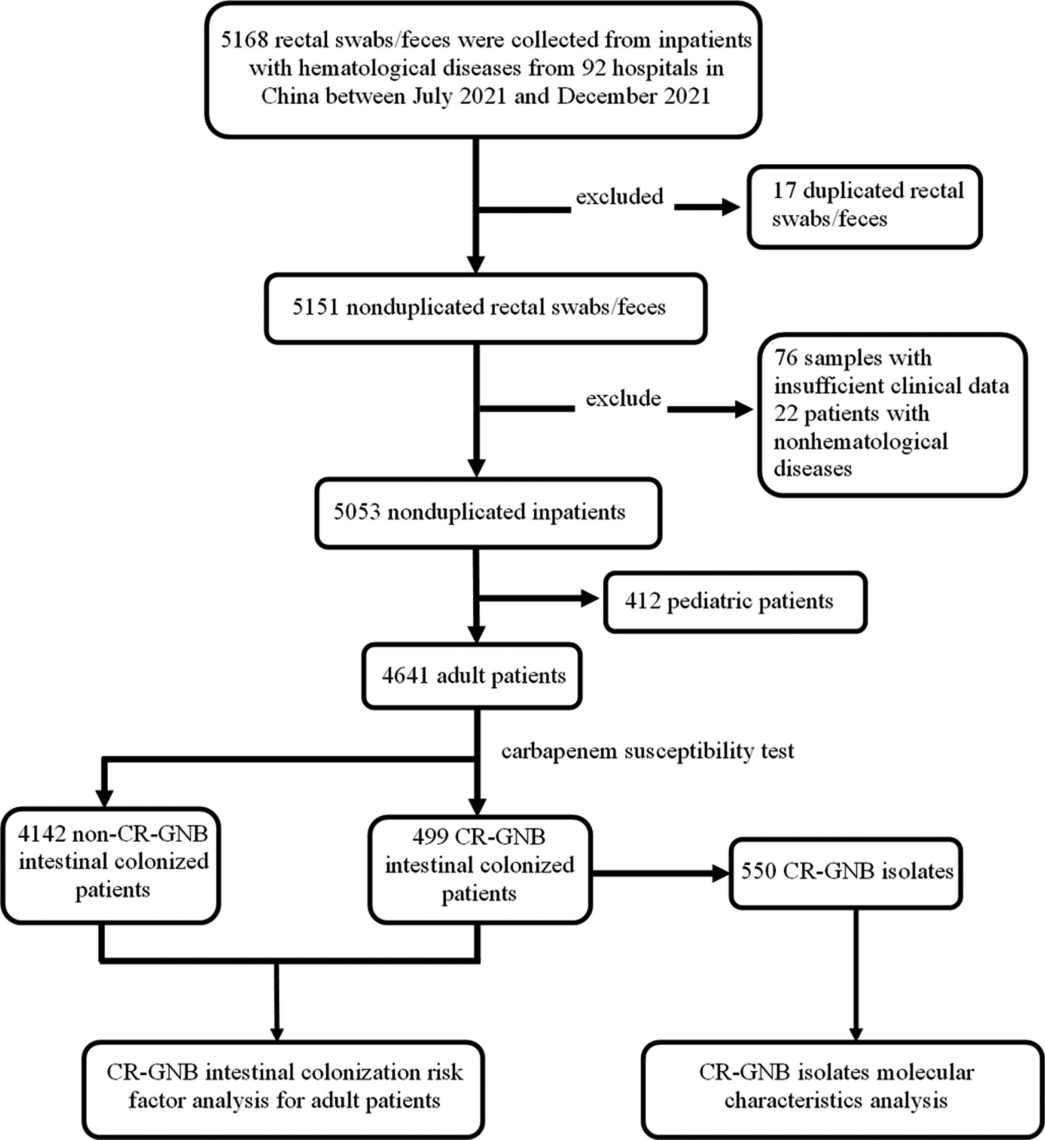
Flowchart showing the recruitment procedure, risk factor analysis of CR-GNB intestinal colonization, and molecular characteristic analysis of CR-GNB isolates.

**Fig 2:**
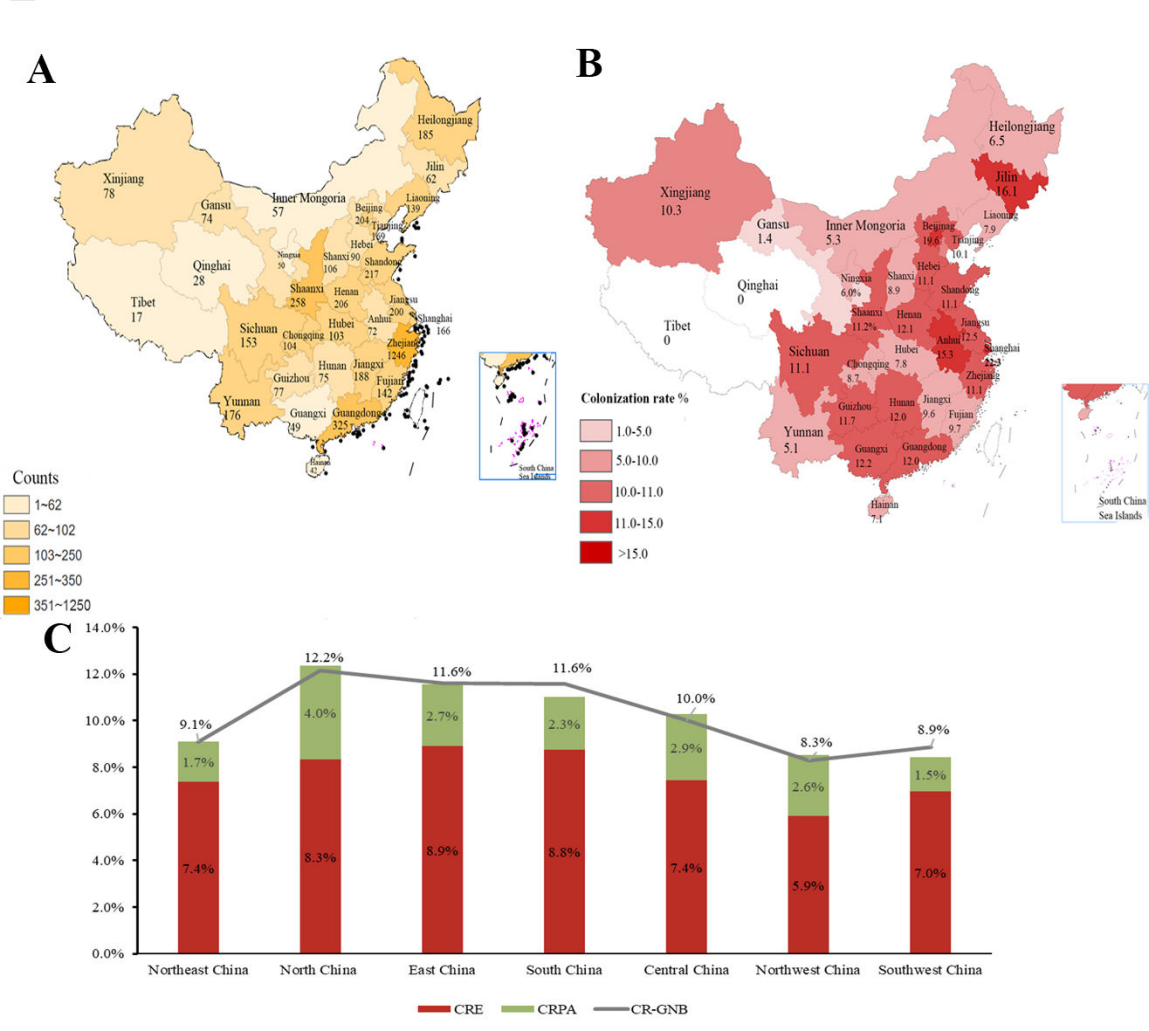
(**A**) The number of screened individuals in various provinces and autonomous regions of China. (**B**) The intestinal CR-GNB colonization rate in each province. A darker color represents a greater number of screened patients or a higher colonization rate. (**C**) The intestinal CRE, CRPA, and CR-GNB colonization rates in each administrative region of China. Red represents the CRE colonization rate; green represents the CRPA colonization rate; and the line segment represents the CR-GNB colonization rate. The maps were generated from the Standard map service website provided by the Center for Mapping Technology Review, Ministry of Natural Resources, People’s Republic of China.

From July 20, 2021, to December 31, a total of 5,053 non-duplicated inpatients from 92 hospitals in China were screened, and 4,641 adult patients (age ≥18) were analyzed as shown in Fig. 1. The number of patients screened from each province is shown in Fig. 2A. Out of 4,641 adult patients, 499 were found to have intestinal colonization with CR-GNB (including CRE, CRPA, or CRAB) isolates, and the colonization rate was 10.8% (499/4,641). Further analysis of the intestinal colonization patients revealed that 376 patients had CRE colonization, 121 had CRPA colonization, and 15 had CRAB colonization. In 448 patients, only one strain was isolated, while in the other 51 patients, two strains were isolated, and the CRE, CRPA, and CRAB intestinal colonization rates in adult patients with hematological diseases were 8.1% (376/4,641), 2.6% (121/4,641), and 0.3% (15/4,641), respectively.

Additionally, the intestinal CR-GNB colonization rate was different in different provinces, as shown in Fig. 2B. The colonization rate was highest in Beijing and Shanghai and lowest in Qinghai and Tibet. Additionally, we analyzed the CRE and CRPA colonization rates in seven geographical regions in China. As shown in Fig. 2C, the CRE colonization rate was higher in East, South, and North China, while the CRPA colonization rate was higher in North and Central China. In total, the intestinal CR-GNB colonization rates in the southeastern and northeastern parts of China were significantly higher than those in the southwestern and northwestern parts.

### Risk factors for CR-GNB intestinal colonization in adult patients

To identify the risk factors for intestinal colonization by CR-GNB isolates, patients were categorized into either the colonization group or the non-colonization group based on the presence or absence of intestinal colonization. Overall, 499 colonized and 4,142 non-colonized patients were included in the analysis. The demographics and characteristics of patients with hematological diseases before screening are shown in Table 1. Both groups showed a male predominance. Acute leukemia accounted for the highest proportion of all hematological diseases.

**TABLE 1:** Characteristics of patients with hematological diseases

Parameters	Total (*n* = 4,641)	Patients with intestinal colonization (*n* = 499)	Patients without intestinal colonization (*n* = 4,142)
Age (median, IQR)	54 (39–65)	52 (37–64)	54 (39–65)
Gender			
Male	2,553 (55.0%)	298 (59.7%)	2,255 (54.4%)
Female	2,088 (45.0%)	201 (40.3%)	1,887 (45.6%)
Diagnosis			
Acute leukemia	2,150 (46.3%)	287 (57.5%)	1,863 (45.0%)
Chronic leukemia	130 (2.8%)	15 (3.0%)	115 (2.8%)
Lymphoma	989 (21.3%)	81 (16.2%)	908 (21.9%)
Myelodysplastic syndromes	390 (8.4%)	42 (8.4%)	348 (8.4%)
Plasma cell dyscrasia	694 (5.0%)	43 (8.6%)	651 (15.7%)
Nonneoplastic hematologic diseases	167 (3.6%)	20 (4.0%)	147 (3.5%)
Undiagnosed patients with abnormal blood cell counts	92 (2.0%)	8 (1.6%)	84 (2.0%)
Diagnosis time			
≥ 6 months	1,951 (42.0%)	223 (44.7%)	1,728 (41.7%)
< 6 montths	2,690 (58.0%)	276 (55.3%)	2,414 (58.3%)
Therapy			
Chemotherapy	3572 (77.0%)	363 (72.7%)	3209 (77.5%)
Hematopoietic stem cell transplantation	591 (12.7%)	98 (19.6%)	493 (11.9%)
Symptomatic treatment	478 (10.3%)	38 (7.6%)	440 (10.6%)
Antimicrobial agents (within 6 months)			
None	1,446 (31.2%)	80 (16.0%)	1,366 (33.0%)
Carbapenems	551 (11.9%)	61 (12.2%)	490 (11.8%)
Other β-lactams (except for carbapenems)	971 (20.9%)	95 (19.0%)	876 (21.1%)
Carbapenems + other β-lactams	1,190 (28.7%)	249 (49.9%)	1,439 (31.0%)
Without any β-lactams	234 (5.0%)	14 (2.8%)	220 (5.3%)
Diabetes			
No	4,287 (92.4%)	467 (93.2%)	3,822 (92.3%)
Yes	354 (7.6%)	32 (6.8%）	320 (7.7%)
Deep vein catheterization			
No	2,392 (51.5%)	221 (44.3%)	2,171 (52.4%)
Yes	2,249 (48.5%)	278 (55.7%)	1,971 (47.6%)
Gastrointestinal signs and symptoms (within 1 week)			
No	4,197 (90.4%)	429 (86%)	3,768 (91%)
Yes	444 (9.6%)	70 (14.0%)	374 (9.0%)
Sign of infections (within 1 week)			
None	3,414 (73.6%)	306 (61.3%)	3,108 (75.0%)
Bloodstream infection	92 (2.0%)	18 (3.6%)	74 (1.8%)
Abdominal infection	37 (0.8%)	11 (2.2%)	26 (0.6%)
Perianal infection	109 (2.3%)	15 (3.0%)	94 (2.3%)
Other infection	989 (21.3%)	149 (29.9%)	840 (20.3%)

As shown in Table 2, after conducting univariate analysis, age, sex, diagnosis, therapy, use of antimicrobial agents, deep vein catheterization, gastrointestinal infection signs and symptoms, and signs of infections were included in the multivariable analysis (*P* < 0.2). When entering the multivariate logistic analysis, male sex (OR 0.820; 95% CI 0.676–0.995, *P* = 0.045), hematopoietic stem cell transplantation (OR 1.421; 95% CI 1.085–1.861, *P* = 0.011), carbapenem (OR 1.574; 95% CI 1.086–2.282, *P* = 0.017), and/or other β-lactam (OR 1.537; 95% CI 1.114–2.119, *P* = 0.009) usage in the 6 months before screening and other infections within 1 week before screening (except for perianal infection) (*P* = 0.008) were more likely to result in CR-GNB intestinal colonization. For different diagnoses of hematological diseases, patients with acute leukemia were more likely to be colonized with CR-GNB isolates compared with lymphoma (OR 0.753; 95% CI 0.574–0.987, *P* = 0.040) and plasma cell dyscrasia patients (OR 0.563; 95% CI 0.394–0.850, *P* = 0.002). The power analysis revealed that the calculated power was 1.0, suggesting that the result was appropriately powered.

**TABLE 2:** Risk factors for intestinal colonization of CR-GNB in patients with hematological diseases

Parameters	Univariate analysis		Multivariate analysis	
	OR (95% CI)	*P* value	OR (95% CI)	*P* value
Age (median, IQR)	0.996 (0.990–1.002)	0.153	1.005 (0.999–1.011)	0.129
Gender				
Male	Ref		Ref	
Female	0.806 (0.667–0.974)	0.025	0.820 (0.676–0.995)	0.045
Diagnosis				
Acute leukemia	Ref		Ref	
Chronic leukemia	0.676 (0.376–1.212)	0.189	0.800 (0.438–1.460)	0.468
Lymphoma	0.583 (0.452–0.752)	< 0.001	0.753 (0.574–0.987)	0.040
Myelodysplastic syndromes	0.790 (0.566–1.103)	0.166	0.823 (0.579–1.172)	0.280
Plasma cell dyscrasia	0.424 (0.304–0.591)	< 0.001	0.563 (0.394–0.850)	0.002
Nonneoplastic hematologic diseases	0.777 (0.469–1.288)	0.328	1.016 (0.590–1.750)	0.954
Undiagnosed patients with abnormal blood cell counts	0.382 (0.138–1.054)	0.063	0.552 (0.193–1.580)	0.268
Diagnosis time				
≥ 6 months	Ref			
< 6 montths	1.103 (0.915–1.331)	0.304		
Therapy				
Chemotherapy	Ref		Ref	
Hematopoietic stem cell transplantation	1.758 (1.379–2.240)	< 0.001	1.421 (1.085–1.861)	0.011
Symptomatic treatment	0.765 (0.540–1.085)	0.133	0.945 (0.642–1.392)	0.775
Antimicrobial agents (within 6 months)				
None	Ref		Ref	
Carbapenems	2.126 (1.500–3.013)	< 0.001	1.574 (1.086–2.282)	0.017
Other β-lactams (except for carbapenems)	1.852 (1.359–2.532)	< 0.001	1.537 (1.114–2.119)	0.009
Carbapenems + other β-lactams	3.537 (2.745–4.650)	< 0.001	2.533 (1.879–3.414)	< 0.001
Without any β-lactams	1.087 (0.605–1.951)	0.781	0.943 (0.522–1.703)	0.846
Diabetes				
No	Ref			
Yes	0.904 (0.629–1.298)	0.585		
Deep vein catheterization				
No	Ref		Ref	
Yes	1.349 (1.119–1.625)	0.002	1.012 (0.828–1.238)	0.906
Gastrointestinal infection signs and symptoms (within 1 week)				
No	Ref		Ref	
Yes	1.657 (1.261–2.176)	<0.001	1.232 (0.923–1.644)	0.156
Sign of infections (within 1 week)				
None	Ref		Ref	
Bloodstream infection	2.862 (1.719–4.765)	< 0.001	1.812 (1.072–3.063)	0.026
Abdominal infection	2.642 (1.419–4.917)	< 0.001	1.922 (1.011–3.654)	0.046
Perianal infection	1.244 (0.675–2.292)	0.483	0.857 (0.459–1.598)	0.627
Other infection	1.847 (1.496–2.281)	< 0.001	1.357 (1.083–1.701)	0.008

Additionally, we compared the risk factor differences between CRE- and CRPA-colonized patients. As shown in Table S1, 376 CRE-colonized patients and 121 CRPA-colonized patients were included in analysis, and the multivariate analysis showed that compared with CRPA-colonized patients, patients using other β-lactams (*P* < 0.001) or carbapenems and other β-lactams (*P* = 0.006) were more likely to be colonized with CRE isolates. (Table S2)

### Distribution of colonized CR-GNB isolates and AST results

A total of 550 CR-GNB isolates were collected from 499 adult patients, including 414 CRE isolates, 121 CRPA isolates, and 15 CRAB isolates. Among CRE isolates, there were 184 carbapenem-resistant *Escherichia coli* (CREC) isolates, 171 carbapenem-resistant *Klebsiella pneumoniae* (CRKP) isolates, 42 carbapenem-resistant *Enterobacter* spp. isolates, nine carbapenem-resistant *K. oxytoca* isolates, seven carbapenem-resistant *K. aerogenes* isolates, and one carbapenem-resistant *Salmonella enterica* serovar Indiana isolate.

The AST results are shown in Table 3. Among CRE isolates, polymyxin B demonstrated favorable susceptibility, with the resistance rate less than 15% (highest of 14.3% in carbapenem-resistant *K. aerogenes* and lowest of 0 in carbapenem-resistant *K. oxytoca*). Tigecycline showed a similarly favorable susceptibility toward CREC, carbapenem-resistant *Enterobacter* spp., and carbapenem-resistant *K. aerogenes*, with the resistance rate below 10%. However, its efficacy diminished against CRKP and carbapenem-resistant *K. aerogenes*, with their resistance rates reaching 32.7% and 28.6%, respectively. Notably, carbapenem-resistant *K. aerogenes* isolates were all susceptible to amikacin and fosfomycin, and the resistance rates for other CRE isolates to these two antimicrobials remained below 25%. The three new β-lactam-β-lactamase inhibitor combinations (BLBLIs), namely, ceftazidime–avibactam, meropenem–vaborbactam, and imipenem–cilastatin–relebactam, all exhibited favorable susceptibility to CRKP isolates, with a resistance rate below 25%. However, their efficacy is significantly reduced against CREC, carbapenem-resistant *Enterobacter* spp. and carbapenem-resistant *K. oxytoca* isolates, with a resistance rate nearly over 70%, except for 67.9% of CREC to meropenem–vaborbactam.

**TABLE 3:** Antimicrobial susceptibility results of CR-GNB isolates .^*b*^^,*c*^

	*E. coli* (*n* = 184)			*K. pneumoniae* (*n* = 171)			Enterobacter spp. (*n* = 42)			K. oxytoca (*n* = 9)			K. aerogenes (*n* = 7)			P. aeruginosa (*n* = 121)			A. baumannii (*n* = 15)		
	%R	MIC_50_	MIC_90_	%R	MIC_50_	MIC_90_	%R	MIC_50_	MIC_90_	%R	MIC_50_	MIC_90_	%R	MIC_50_	MIC_90_	%R	MIC_50_	MIC_90_	%R	MIC_50_	MIC_90_
ETP	100	32	128	100	64	>128	100	32	64	100	16	128	100	64	>128	-	-	-	-	-	-
IMP	84.2	4	16	81.3	8	64	85.7	4	32	88.9	4	32	100	16	64	100	16	32	100	32	128
MEM	92.4	8	32	92.4	8	>128	88.1	8	32	100	8	64	85.7	16	64	41.3	4	16	93.3	32	64
CAZ	98.9	>128	>128	97.1	>128	>128	95.2	>128	>128	100	>128	>128	71.4	64	>128	18.2	4	64	-	-	-
FEP	100	>128	>128	92.4	>128	>128	92.9	128	>128	88.9	64	>128	28.6	4	>128	12.4	4	32	-	-	-
AZT	78.3	64	>128	93.6	>128	>128	69.0	64	>128	88.9	>128	>128	71.4	16	>128	23.1	8	64	-	-	-
CIP	89.7	64	>128	95.3	>128	>128	85.7	8	128	66.7	4	>128	71.4	8	32	9.9	0.25	1	66.7	>32	>32
LEV	85.9	32	64	90.1	64	>128	81.0	8	64	66.7	4	128	71.4	8	16	14.0	0.5	4	-	-	-
FOS^*a*^	22.3	2	512	15.2	128	256	7.1	32	128	11.1	16	512	0	64	64	7.4	64	128	-	-	-
AK	16.8	4	128	24.6	4	>256	4.8	2	16	11.1	2	>256	0	1	4	0.8	4	8	66.7	>256	>256
PTZ	98.4	>128/4	>128/4	96.5	>128/4	>128/4	88.1	>128/4	>128/4	88.9	>128/4	>128/4	100	>128/4	>128/4	17.4	8/4	>128/4	-	-	-
F/S (2:1) ^a^	98.9	>128/64	>128/64	97.1	>128/64	>128/64	92.9	>128/64	>128/64	100	>128/64	>128/64	57.1	64/32	>128/64	20.7	16/8	64/8	73.3	64/32	128/64
F/S (1:1)	-	-	-	-	-	-	-	-	-	-	-	-	-	-	-	-	-	-	73.3	32/32	64/64
CZA	79.9	>128/4	>128/4	24.0	2/4	>128/4	88.1	>128/4	>128/4	88.9	>128/4	>128/4	28.6	2/4	>128/4	7.4	2/4	8/4	-	-	-
MEV	67.9	16/8	64/8	19.9	2/8	64/8	73.8	16/8	64/8	77.8	16/8	64/8	71.4	16/8	64/8	28.1	4/8	16/8	-	-	-
I/R	81.5	8/4	64/4	21.1	1/4	16/4	85.7	16/4	32/4	88.9	16/4	64/4	71.4	16/4	64/4	9.1	1/4	4/4	-	-	-
C/T	98.9	>128/4	>128/4	95.3	128/4	>128/4	95.2	>128/4	>128/4	100	>128/4	>128/4	57.1	8/4	>128/4	5.8	1/4	4/4	-	-	-
POL	10.3	1	4	8.2	1	2	9.5	1	2	0	1	1	14.3	1	16	0	2	2	0	1	2
TGC	0.5	0.125	1	32.7	4	16	7.1	0.5	4	0	0.5	4	28.6	2	16	-	-	-	0	0.25	0.5

^*a*^ The MIC breakpoints of CRE and CRPA to fosfomycin refer to breakpoints applied to *E. coli* urinary tract isolates; the MIC breakpoints of cefoperazone–sulbactam refer to breakpoints applied to cefoperazone.

^*b*^ %R, resistance rate; MIC_50/90_, 50%/90% minimum inhibitory concentration (μg/mL); -, represents antimicrobial susceptibility testing not performed.

^*c*^ ETP, ertapenem; IMP, imipenem; MEM, meropenem; CAZ, ceftazidime; FEP, cefepime; AZT, aztreonam; CIP, ciprofloxacin; LEV, levofloxacin; FOS, fosfomycin; AK, amikacin; PTZ, piperacillin–tazobactam; F/S, cefoperazone–sulbactam; CZA, ceftazidime–avibactam; MEV, meropenem–vaborbactam; I/R, imipenem–cilastatin–relebactam; C/T, ceftolozane-tazobactam; POL, polymyxin B; TGC, tigecycline.

For CRPA isolates, all the antipseudomonal agents exhibited favorable antimicrobial activity, with resistance rates less than 30%. Moreover, in CRAB isolates, only polymyxin B and tigecycline showed low resistance rates.

### Antimicrobial resistance genes and phylogenetic analysis

**Fig 3:**
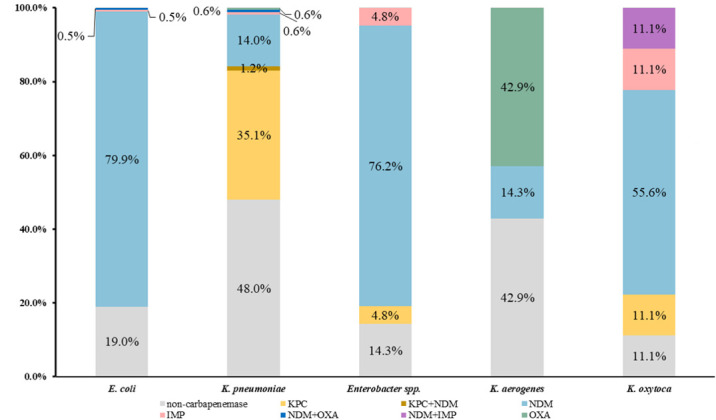
Carbapenemase distribution in CRE isolates. Gray represents non-carbapenemase isolates. Yellow represents KPC-producing isolates. Brown represents isolates producing both KPC and NDM. Light blue represents NDM-producing isolates. Pink represents IMP-producing isolates. Dark blue represents isolates producing both NDM and OXA. Purple represents isolates producing both NDM and IMP. Green represents OXA-producing isolates. The number represents the exact portion of each carbapenemase in each CRE species.

Carbapenemase production was observed in 288 out of 414 CRE isolates, which accounted for 69.6% of all CRE isolates (288/414). Among these carbapenemase-producing isolates, NDM was the predominant type and accounted for 73.0% of all isolates (210/288). KPC was detected in 63 CRE isolates and accounted for 21.9% (63/288) of all carbapenemases. The remaining isolates were positive for IMP (2.1%, 6/288) and OXA (1.4%, 4/288). In addition, there were two CRKP isolates that produced both KPC and NDM, one carbapenem-resistant *K. oxytoca* isolate that produced both IMP and NDM, and one CRKP isolate and one CREC isolate that produced both NDM and OXA. The carbapenem-resistant *Salmonella enterica* serovar Indiana isolate produced NDM-9. As shown in Fig. 3, the production of carbapenemases varied significantly among different CRE isolate species. NDM was overwhelmingly dominant in CREC (79.9%) and carbapenem-resistant *Enterobacter* spp. (76.2%) isolates. Among the CRKP isolates, KPC (35.1%) was the prevailing carbapenemase, while NDM (14.0%) also accounted for a significant portion of carbapenemases.

Among CRPA isolates, carbapenemase production was detected in only six isolates (5.0%, 6/121). Among these, four isolates produced KPC-2, one isolate produced VIM-2, and one isolate produced both KPC-2 and AFM-1. Among the CRAB isolates, nine isolates produced both OXA-23 and OXA-66, and two isolates produced NDM-1 and OXA-58 or OXA-72. The remaining four isolates produced OXA-72, OXA-72 and OXA-729; OXA-23 and OXA-64; OXA-510; and OXA-58, respectively (Tables S8 and S9).

**Fig 4:**
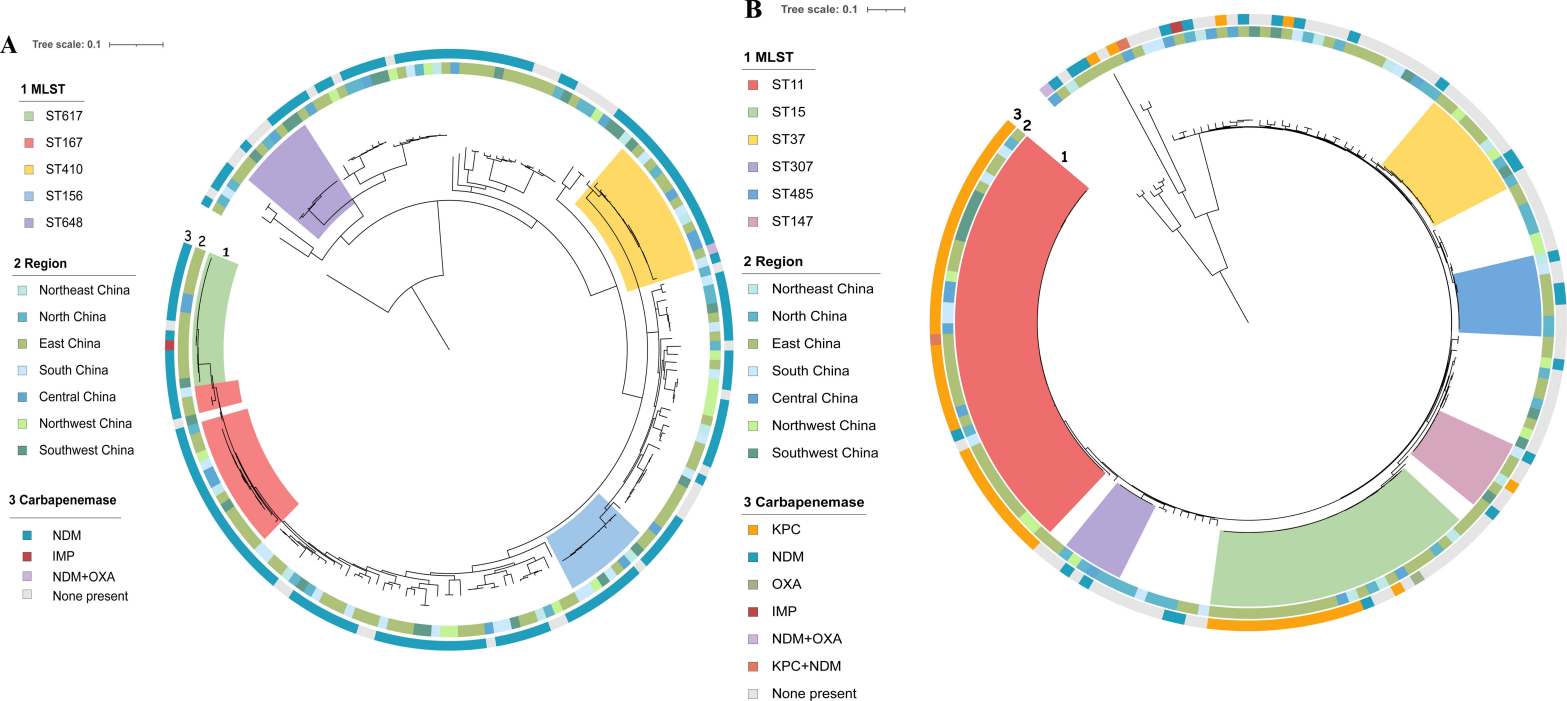
Phylogenetic population structures: (**A**) 184 CREC isolates. (**B**) 171 CRKP isolates. The phylogenetic trees were rooted in the midpoint. MLST, multilocus sequence type. In circle 1, the unlabeled isolates represent scattered STs (*n* < 5) (see supplementary tables). Circle 2 represents the region from which patient isolates were collected. Circle 3 represents the carbapenemase type produced by the isolates.

Phylogenetic analysis was performed on CREC and CRKP isolates, as shown in Fig. 4. ST167, ST410, and ST617 accounted for 9.8% (18/184), 8.7% (16/184), and 8.2% (15/184) of all CREC isolates, respectively. NDM carbapenemase was found in each ST and region in China, and clonal spread was observed. In CRKP isolates, ST11 and ST15 were the most abundant and accounted for 24.6% (42/171) and 14.6% (25/171) of all CRKP isolates, respectively. KPC carbapenemase was mostly detected in ST11 and ST15, and clonal spread was observed. In other CR-GNB isolates, the MLST results showed that they had discrete STs (Tables S3 to S7).

## DISCUSSION

This multicenter case-control study was conducted in China and involved specimens from 31 provinces. The results revealed that the intestinal colonization rate of CR-GNB in adult patients with hematological diseases in China was 10.8%. Comparing these findings with reports from European countries, the colonization rate in China was higher than the reported rate of 3%–4% in Europe (12, 13). However, the rate in China was lower than the rate of 75.5% reported in India (14). Additionally, single-center studies in China have reported CRE colonization rates ranging from 6.6% to 23.8% in patients with hematologic malignancies (15, 16). Among all colonized CR-GNB isolates, CRE colonization accounted for the highest, followed by CRPA, while CRAB colonization was rare. This was different from ICU patients, in whom CRKP and CRAB were the most commonly colonized pathogens (17, 18). Moreover, metalloenzymes, especially NDM, produced in CRE isolated from patients with hematological diseases were significantly higher than the isolates separated from the whole population. In our study, the portion of NDM was 79.9% and 76.2% in CREC and carbapenem-resistant *Enterobacter* spp., respectively, much higher than the result of resistance surveillance from 2015 to 2016 in China, which reported that NDM was the predominant carbapenemase in CREC (52.1%) and carbapenem-resistant *Enterobacter* spp. (50.3%) (19). In CRKP, although KPC was still the predominant carbapenemase (35.1%), the portion of NDM (14%) elevated significantly compared to the isolates from the ICU (20, 21). CRKP strains also had a high percentage of non-carbapenemase-producing strains (up to 48%). The carbapenem MICs of the non-carbapenemase-producing strains were lower than those of carbapenemase-producing strains. These strains were all producing ESBL enzymes or AmpC enzymes, and the possible carbapenem resistance mechanism for these non-carbapenemase-producing strains may be high production of ESBLs and AmpC enzymes together with inactivation of pore proteins. This study also found that the colonization rate fluctuated in different regions in China, ranging from 0% to 20.0%. These findings highlight the need for CR-GNB intestinal colonization screening in patients with hematological diseases in each region. Epidemiology data can aid in selecting appropriate empirical treatments and implementing measures to control the spread of CR-GNB isolates.

Identifying the risk factors for CR-GNB colonization can aid in determining whether patients are at high risk, which enables healthcare facilities to selectively implement proactive screening measures. However, studies on the risk factors for CR-GNB colonization in patients with hematological diseases are inadequate in China. Cao *et al.* found that pulmonary infection, perianal infection, and carbapenem application in the 3-month pretransplant were independent risk factors for CRE colonization in HSCT recipients (16). Jaiswal *et al.* found that acute leukemia and previous long-term hospitalization were independent risk factors for CRE colonization in patients with hematological malignancies in a cohort study (22). In an Italian multicenter study, less age (< 60 years), non-autologous HSCT, and urinary tract catheterization were risk factors for CR-GNB intestinal colonization in patients with hematological diseases (13). Our study showed that in adult patients with hematological diseases, male sex (*P* = 0.045), acute leukemia (*P* = 0.048), HSCT (*P* = 0.011), carbapenem (*P* = 0.017), and/or other β-lactam (*P* = 0.009) usage in the 6 months before screening and other infections within 1 week before screening (except for perianal infection) (*P* = 0.008) were risk factors for CR-GNB intestinal colonization. Most patients with acute leukemia need HSCT therapy, and HSCT can cause mucosal toxicity and leads to increased permeability in the gastrointestinal tract, prolonged hospitalization, and use of antibiotics (7). Previous infection also leads to antibiotic usage. Antibiotic usage may disrupt the gastrointestinal microflora and eradicate susceptible competing strains, thus promoting CR-GNB colonization. Moreover, our study compared the risk factor difference between CRE- and CRPA-colonized patients. The result showed using of other β-lactams or carbapenem and other β-lactams was more likely to cause CRE intestinal colonization. A previous study reported that reintroduction of *E. coli* in antibiotic-treated mice increases defense against *P. aeruginosa* colonization (23), while the interaction between CRE and CRPA in the intestine needs to be further explored.

Furthermore, our study also clarifies the molecular characteristics of CR-GNB isolates. Phylogenetic analysis results showed the extreme divergence of CREC and CRKP strains from patients with hematological diseases. Clonal transmission could be observed, but unlike CRE strains isolated from the general population, the proportion of traditional dominant clones, such as ST11, among CRKP isolates decreased significantly (19, 24). This may suggest that original *Enterobacterales* strains colonized in the intestine undergo evolution under the selective pressure of antimicrobial agents. And, in recent years, the rate of carbapenemase-producing CRPA has gradually increased (20). Globally, the most common carbapenemases in CRPA are metalloenzymes, including VIM, IMP, and NDM (21). However, some studies in Zhejiang Province reported that KPC-2-producing CRPA accounted for 21.1%–40.4% of all isolates, while ST463-CRPA accounted for 43.8%–70.9% of KPC-2-producing isolates (25, 26). In our study, the proportion of carbapenemase-producing CRPA was 5.0% (6/121). Notably, five of the isolates produced KPC-2 and belonged to ST463; these strains were all isolated from Zhejiang Province. Recent studies showed that KPC-2-producing ST463-CRPA carries both type III secretion systems, ExoU and ExoS, indicating the coexistence of resistance and hypervirulence and may be found in emerging high-risk clones (27, 28).

There are still some limitations to this study. First, although this study included specimens that were collected across the country, the number of specimens differed in each province, which may have resulted in bias. Second, although this study included many patients, certain variables, such as neutropenia and previous ICU admission, were not evaluated. These factors may also influence the assessment of risk.

In conclusion, in this study, we analyzed the risk factors for intestinal colonization by CR-GNB isolates in patients with hematological diseases by performing a multicenter case‒control study. The CR-GNB intestinal colonization rate was high in patients with hematological diseases in China, among which CRE was the main pathogen. The proportion of colonized CRE isolates with metallo-β-lactamases was high, followed by CRPA, while the CRAB colonization was rare. Thus, active screening is important for patients with hematological diseases.

## MATERIALS AND METHODS

### Study design and participants

This was an observational multicenter study conducted from July 20, 2021, to December 31, 2021, in 92 hospitals across China. This study aimed to screen inpatients with hematological diseases for intestinal colonization by CR-GNB. Rectal swabs or feces were collected from all inpatients of the hematology ward at certain hospitals on specific days (Table S10), with no age or diagnosis exclusion. As shown in Fig. 1, a total of 4,641 adult patients were included.

All samples were sent to the central laboratory and cultured in trypticase soy broth (TSB) at 37°C overnight. The following day, the cultured TSB samples were inoculated on specific media for the identification of different species, including *E. coli*/coliform, *Acinetobacter* chromogenic media (CHROMagar, France), *P. aeruginosa* selection medium (Sangon Biotech, China), and *K. pneumoniae* selection medium (Hopebio, China). Monoclonal colonies that were selected were further analyzed using MicroTyper MS (Skyray Instruments, China) to determine the exact species. Confirmed monoclonal colonies were saved for carbapenem susceptibility testing.

### Statistical analysis

The clinical data of all patients were collected retrospectively, including (1) general information such as age and gender and (2) risk factors including the diagnosis of hematological disease, time of illness, treatment within 6 months (chemotherapy, HSCT, or only symptomatic and supportive treatment), antimicrobial agent use within 6 months, diabetes, deep vein catheterization, gastrointestinal illness symptoms (abdominal pain, diarrhea, and gastrointestinal bleeding) within 1 week, and signs of infections (blood, perianal, abdominal cavity, or other parts) within 1 week.

Data were analyzed using SPSS 26.0 (IBM Corporation). Binary logistic regression analysis was used, and the results included odds ratios (ORs) and 95% confidence intervals (CIs). Variables with *P* < 0.2 in univariate analysis were included in multivariate analysis, and *P* < 0.05 was considered statistically significant. The GPower (v3.1.9.7) was used to do a *post hoc* power analysis. And *t*-test and linear multiple regression fixed model was used. The total sample size was 4641, the α error was 0.05, and the effect size was 0.15.

### Antimicrobial susceptibility testing (AST)

The AST of carbapenems was performed using the agar dilution method. For CRE and CRPA isolates, AST of other antimicrobial agents (including ceftazidime, cefepime, aztreonam, ciprofloxacin, levofloxacin, fosfomycin, amikacin, piperacillin–tazobactam, cefoperazone–sulbactam (2:1), ceftazidime–avibactam, meropenem–vaborbactam, imipenem–cilastatin–relebactam, ceftolozane–tazobactam, and polymyxin B) was performed by the broth microdilution method using customized susceptibility plates (Thermo Fisher Scientific, USA). Additionally, for CRE isolates, AST of tigecycline was performed using the broth microdilution method. For CRAB isolates, AST of other antimicrobial agents (including ciprofloxacin, amikacin, cefoperazone–sulbactam (in both 2:1 and 1:1 ratios), polymyxin B, and tigecycline) was performed by the broth microdilution method using customized susceptibility plates (Thermo Fisher Scientific, USA). *Escherichia coli* ATCC 25922 and *Pseudomonas aeruginosa* ATCC 27853 were used for quality control. The results for tigecycline were interpreted according to the criteria proposed by the US Food and Drug Administration (FDA, https://www.fda.gov/drugs/development-resources/tigecycline-injection-products), while those for other antimicrobial agents were interpreted following Clinical and Laboratory Standards Institute (CLSI) guidelines (29). For results of the resistance rate, we recommend that less than 25% were low resistance rate and higher than 75% were high resistance rate.

### Whole-genome sequencing

Genomic DNA of all CR-GNB isolates was extracted using a QIAamp DNA Mini Kit (Qiagen, Hilden, Germany). Short-read sequencing (Illumina, San Diego, USA) and genome assembly were performed *de novo* using shovill v0.9.0 (https://github.com/tseemann/shovill). Multilocus sequence typing (MLST) results were analyzed using mlst v2.19.0 (https://github.com/tseemann/mlst), and antimicrobial resistance genes were analyzed using ABRicate v1.0.0 (https://github.com/tseemann/abricate) with the NCBI database. Prokka v1.14.6 (https://github.com/tseemann/prokka) was used for open-reading frame (ORF) annotation. Alignment of core genes from all CREC and CRKP isolates was achieved with panaroo v.1.2.7 (https://github.com/gtonkinhill/panaroo). The maximum-likelihood phylogenetic trees of CREC and CRKP isolates were inferred by IQ-TREE v2.1.2 using the best-fit model according to BIC (http://www.iqtree.org/). The phylogenetic tree was visualized using iTOL v6 (https://itol.embl.de/).

## Data Availability

The BioProject number under which the generated sequencing data can be found is PRJNA1029722.

## SUPPLEMENTAL MATERIAL

Supplemental material spectrum.04299-23-s0001.docx Tables S1 to S10.

ASM does not own the copyrights to Supplemental Material that may be linked to, or accessed through, an article. The authors have granted ASM a non-exclusive, world-wide license to publish the Supplemental Material files. Please contact the corresponding author directly for reuse.
