# Supplementary material for: Risk factors and molecular epidemiology of intestinal colonization by carbapenem-resistant Gram-negative bacteria in patients with hematological diseases: a multicenter case‒control study
Source: Microbiol Spectr. 2024 Jun 7;12(7):e04299-23. doi: 10.1128/spectrum.04299-23 (PMC11218473; doi:10.1128/spectrum.04299-23)
Supplement: Supplemental material — Tables S1 to S10. [file spectrum.04299-23-s0001.docx]

**Table S1 Characteristics of patients with intestinal colonization of CRE or CRPA isolates.**

| Parameters | Patients colonized with CRE  (n = 376) | Patients colonized with CRPA  (n = 121) |
| --- | --- | --- |
| **Age**  (Medium, IQR) | 53 (37-64) | 50 (35-64) |
| **Gender** |  |  |
| Male | 216 (57.4%) | 75 (62.0%) |
| Female | 160 (42.6%) | 46 (38.0%) |
| **Diagnosis** |  |  |
| Acute leukemia | 213 (56.6%) | 75 (62.0%) |
| Chronic leukemia | 8 (2.1%) | 5 (4.1%) |
| Lymphoma | 66 (17.6%) | 17 (14.0%) |
| Myelodysplastic Syndromes | 38 (10.1%) | 8 (6.6%) |
| Plasma cell dyscrasia | 32 (8.5%) | 11 (9.1%) |
| Nonneoplastic hematologic diseases | 15 (4.0%) | 4 (3.3%) |
| Undiagnosed patients with  abnormal blood cell counts | 4 (1.1%) | 1 (0.8%) |
| **Diagnosis time** |  |  |
| ≥ 6 months | 160 (42.6%) | 59 (48.8%) |
| < 6 montths | 216 (57.4%) | 62 (51.2%) |
| **Therapy** |  |  |
| Chemotherapy | 278 (73.9%) | 86 (71.1%) |
| Hematopoietic stem cell transplantation | 68 (18.1%) | 27 (22.3%) |
| Symptomatic treatment | 30 (8.0%) | 8 (6.6%) |

**(continue)**

| **Antimicrobial agents (within 6 months)** |  |  |
| --- | --- | --- |
| None | 47 (12.5%) | 33 (27.3%) |
| Carbapenems | 42 (11.2%) | 17 (14.0%) |
| Other β-lactams (except for carbapenems) | 83 (22.1%) | 10 (8.3%) |
| Carbapenems + other β-lactams | 192 (51.1%) | 59 (48.8%) |
| without any β-lactams | 12 (3.2%) | 2 (1.7%) |
| **Diabetes** |  |  |
| No | 352 (93.6%) | 112 (92.6%) |
| Yes | 24（6.4%） | 9 (7.4%) |
| **Deep vein catheterization** |  |  |
| No | 169 (44.9%) | 61 (50.4%) |
| Yes | 207 (55.1%) | 60 (49.6%) |
| **Gastrointestinal signs and symptoms (within 1 week)** |  |  |
| No | 321 (85.4%) | 107 (88.4%) |
| Yes | 55 (14.6%) | 14 (11.6%) |
| **Sign of infections (within 1 week)** |  |  |
| none | 220 (58.5%) | 83 (68.6%) |
| Bloodstream infection | 16 (4.3%) | 3 (2.5%) |
| Abdominal infection | 10 (2.7%) | 3 (2.5%) |
| Perianal infection | 10 (2.7%) | 2 (1.7%) |
| Other infection | 120 (31.9%) | 30 (24.8%) |

**Table S2 Risk factors for the intestinal colonization of CRPA compared with CRE.**

| Parameters | Univariate analysis | | Multivariate analysis | |
| --- | --- | --- | --- | --- |
|  | OR (95% CI) | *P* value | OR (95% CI) | *P* value |
| **Age**  (Medium, IQR) | 0.998(0.986-1.010) | 0.706 |  |  |
| **Gender** |  |  |  |  |
| Male | Ref |  | Ref |  |
| Female | 0.828(0.544-1.260) | 0.379 | 0.788(0.506-1.227) | 0.292 |
| **Diagnosis** |  |  |  |  |
| Acute leukemia | Ref |  | Ref |  |
| Chronic leukemia | 1.775(0.563-5.594) | 0.327 | 1.205(0.347-4.187) | 0.770 |
| Lymphoma | 0.732(0.404-1.326) | 0.303 | 0.624(0.326-1.195) | 0.155 |
| Myelodysplastic Syndromes | 0.598(0.267-1.339) | 0.211 | 0.549(0.234-1.285) | 0.167 |
| Plasma cell dyscrasia | 0.976(0.469-2.034) | 0.949 | 0.718(0.317-1.628) | 0.428 |
| Nonneoplastic hematologic diseases | 0.757(0.244-2.354) | 0.631 | 0.652(0.187-2.278) | 0.503 |
| Undiagnosed patients with  abnormal blood cell counts | 0.710(0.078-6.453) | 0.761 | 0.760(0.073-7.872) | 0.818 |
| **Diagnosis time** |  |  |  |  |
| ≥ 6 months | Ref |  | Ref |  |
| < 6 montths | 0.778(0.516-1.174) | 0.232 | 0.827(0.520-1.315) | 0.422 |
| **Therapy** |  |  |  |  |
| Chemotherapy | Ref |  | Ref |  |
| Hematopoietic stem cell transplantation | 1.284(0.773-2.131) | 0.335 | 1.384(0.763-2.509) | 0.285 |
| Symptomatic treatment | 0.862(0.381-1.950) | 0.722 | 0.768(0.294-2.005) | 0.590 |

**(continue）**

| **Antimicrobial agents (within 6 months)** |  |  |  |  |
| --- | --- | --- | --- | --- |
| None | Ref |  | Ref |  |
| Carbapenems | 0.576(0.281-1.182) | 0.133 | 0.572(0.254-1.284) | 0.176 |
| Other β-lactams (except for carbapenems) | 0.172(0.078-0.379) | <0.001 | 0.175(0.076-0.400) | <0.001 |
| Carbapenems + other β-lactams | 0.438(0.257-0.745) | 0.002 | 0.414 (0.220-0.780) | 0.006 |
| without any β-lactams | 0.250(0.052-1.198) | 0.083 | 0.247(0.049-1.230) | 0.088 |
| **Diabetes** |  |  |  |  |
| No | Ref |  |  |  |
| Yes | 1.179 (0.532-2.610) | 0.685 |  |  |
| **Deep vein catheterization** |  |  |  |  |
| No | Ref |  | Ref |  |
| Yes | 0.803(0.533-1.210) | 0.295 | 0.746(0.467-1.190) | 0.218 |
| **Gastrointestinal signs and symptoms (within 1 week)** |  |  |  |  |
| No | Ref |  | Ref |  |
| Yes | 0.764(0.408-1.428) | 0.399 | 0.851(0.433-1.673) | 0.639 |
| **Sign of infections (within 1 week)** |  |  |  |  |
| none | Ref |  | Ref |  |
| Bloodstream infection | 0.497(0.141-1.750) | 0.276 | 0.599(0.163-2.202) | 0.440 |
| Abdominal infection | 0.795(0.214-2.961) | 0.733 | 1.220(0.302-4.935) | 0.780 |
| Perianal infection | 0.530(0.114-2.470) | 0.419 | 0.609(0.122-3.038) | 0.545 |
| Other infection | 0.663(0.413-1.0) | 0.088 | 0.781(0.463-1.317) | 0.354 |

**Table S3 Carbapenemase and STs of intestinal colonized CREC isolates**

| **No.** | **Isolate_ID** | **Carbapenemase** | **ST** |
| --- | --- | --- | --- |
| 1 | CDE0101 | NDM-5 | ST224 |
| 2 | CDE0102 | NDM-5 | ST683 |
| 3 | CDE0104 | NDM-5 | ST5229 |
| 4 | CDE0105 | NDM-5 | ST2179 |
| 5 | CDE0201 | NDM-1 | ST5912 |
| 6 | CDE0202 | IMP-4 | ST617 |
| 7 | CDE0203 | NDM-1 | ST393 |
| 8 | CDE0307 | NDM-5 | ST156 |
| 9 | CDE0404 | / | ST43 |
| 10 | CDE0406 | NDM-5 | ST165 |
| 11 | CDE0501 | NDM-5 | ST770 |
| 12 | CDE0801 | NDM-5 | ST167 |
| 13 | CDE1001 | NDM-5 | - |
| 14 | CDE1003 | NDM-1 | ST410 |
| 15 | CDE1101 | NDM-5 | ST410 |
| 16 | CDE1201 | NDM-13 | ST345 |
| 17 | CDE1203 | NDM-5 | ST457 |
| 18 | CDE1204_1 | NDM-5 | ST167 |
| 19 | CDE1204_2 | NDM-5 | ST167 |
| 20 | CDE1205 | NDM-5 | ST167 |
| 21 | CDE1301 | NDM-5 | - |
| 22 | CDE1302 | / | ST746 |
| 23 | CDE1303 | NDM-5 | ST617 |
| 24 | CDE1401 | NDM-5 | ST354 |
| 25 | CDE1602 | / | ST405 |
| 26 | CDE1701 | NDM-5 | ST69 |
| 27 | CDE1702 | / | ST38 |
| 28 | CDE1801 | / | ST648 |
| 29 | CDE1901 | / | ST648 |
| 30 | CDE1902 | / | ST648 |
| 31 | CDE2001 | / | ST617 |
| 32 | CDE2502 | NDM-1 | ST69 |
| 33 | CDE2504 | NDM-9 | ST156 |
| 34 | CDE2702 | / | ST405 |
| 35 | CDE2704 | NDM-5 | - |
| 36 | CDE2901 | NDM-5 | ST617 |
| 37 | CDE2903 | NDM-5 | ST167 |
| 38 | CDE3201 | NDM-5 | ST167 |
| 39 | CDE3301 | NDM-5 | - |
| 40 | CDE3302 | NDM-5 | ST90 |
| 41 | CDE3304 | NDM-5 | ST410 |
| 42 | CDE3401 | NDM-5 | ST617 |
| 43 | CDE3402_1 | NDM-5 | ST38 |
| 44 | CDE3402_2 | NDM-5 | ST617 |
| 45 | CDE3502 | NDM-5 | ST648 |
| 46 | CDE3602 | NDM-5 | ST410 |
| 47 | CDE3603_1 | NDM-5 | ST617 |
| 48 | CDE3603_2 | NDM-5 | ST617 |
| 49 | CDE3604_1 | NDM-5 | ST617 |
| 50 | CDE3604_2 | NDM-5 | ST617 |
| 51 | CDE3605_1 | NDM-5 | ST167 |
| 52 | CDE3605_2 | NDM-5 | ST167 |
| 53 | CDE3606_1 | NDM-5 | ST38 |
| 54 | CDE3606_2 | NDM-5 | ST617 |
| 55 | CDE3607 | NDM-5 | ST48 |
| 56 | CDE3701 | NDM-5 | ST410 |
| 57 | CDE3704 | NDM-5 | ST48 |
| 58 | CDE3705 | NDM-5 | ST540 |
| 59 | CDE3707 | NDM-5 | ST744 |
| 60 | CDE3709 | NDM-6 | ST361 |
| 61 | CDE3710 | NDM-5 | ST617 |
| 62 | CDE3902 | / | ST361 |
| 63 | CDE3907 | NDM-5 | ST410 |
| 64 | CDE3908 | NDM-5 | ST156 |
| 65 | CDE4001_2 | / | ST648 |
| 66 | CDE4002_1 | NDM-5 | ST167 |
| 67 | CDE4002_2 | NDM-5 | ST167 |
| 68 | CDE4005 | NDM-5 | ST410 |
| 69 | CDE4012 | NDM-5 | ST617 |
| 70 | CDE4013 | NDM-5 | ST617 |
| 71 | CDE4101 | NDM-13 | ST5229 |
| 72 | CDE4201 | NDM-5 | ST555 |
| 73 | CDE4202_1 | NDM-5 | ST744 |
| 74 | CDE4202_2 | NDM-5 | ST744 |
| 75 | CDE4301 | NDM-5 | ST5909 |
| 76 | CDE4303 | NDM-5 | ST226 |
| 77 | CDE4402 | NDM-5 | ST641 |
| 78 | CDE4403 | NDM-5 | - |
| 79 | CDE4404 | NDM-5 | ST43 |
| 80 | CDE4406 | NDM-5 | ST58 |
| 81 | CDE4501 | NDM-5 | ST410 |
| 82 | CDE4503 | / | ST1721 |
| 83 | CDE4703 | NDM-1 | ST457 |
| 84 | CDE4801 | NDM-5 | ST167 |
| 85 | CDE4802_1 | NDM-5 | ST361 |
| 86 | CDE4802_2 | NDM-5 | ST361 |
| 87 | CDE4804 | NDM-5 | ST410 |
| 88 | CDE4805 | NDM-5 | ST167 |
| 89 | CDE4808 | NDM-5 | ST224 |
| 90 | CDE4901 | / | ST46 |
| 91 | CDE4902 | NDM-5 | ST448 |
| 92 | CDE4903 | / | ST405 |
| 93 | CDE4905 | / | ST448 |
| 94 | CDE5001 | OXA-18、NDM-5 | ST410 |
| 95 | CDE5103 | NDM-5 | ST354 |
| 96 | CDE5201 | NDM-13 | - |
| 97 | CDE5301 | NDM-4 | ST457 |
| 98 | CDE5302 | NDM-5 | ST224 |
| 99 | CDE5501 | / | ST156 |
| 100 | CDE5506 | NDM-1 | ST2067 |
| 101 | CDE5601 | / | - |
| 102 | CDE5602 | NDM-5 | ST6969 |
| 103 | CDE5603 | NDM-5 | ST6969 |
| 104 | CDE5605 | NDM-5 | ST156 |
| 105 | CDE5701 | NDM-5 | ST167 |
| 106 | CDE5702 | NDM-5 | ST156 |
| 107 | CDE5703 | NDM-5 | ST354 |
| 108 | CDE5802 | / | ST117 |
| 109 | CDE5806 | / | ST405 |
| 110 | CDE5901 | NDM-5 | ST156 |
| 111 | CDE5902 | NDM-5 | ST156 |
| 112 | CDE5903 | NDM-5 | ST410 |
| 113 | CDE5905 | NDM-5 | ST156 |
| 114 | CDE6001 | NDM-5 | ST167 |
| 115 | CDE6101 | NDM-1 | ST10 |
| 116 | CDE6102 | NDM-5 | ST2973 |
| 117 | CDE6107 | NDM-1 | ST93 |
| 118 | CDE6108 | NDM-5 | ST167 |
| 119 | CDE6109 | NDM-5 | ST167 |
| 120 | CDE6115 | NDM-5 | ST1114 |
| 121 | CDE6117 | / | ST457 |
| 122 | CDE6118 | NDM-5 | ST38 |
| 123 | CDE6202 | NDM-5 | ST1249 |
| 124 | CDE6203 | NDM-5 | ST448 |
| 125 | CDE6204_1 | NDM-5 | ST2705 |
| 126 | CDE6204_2 | NDM-5 | ST2705 |
| 127 | CDE6208 | NDM-5 | ST354 |
| 128 | CDE6209 | NDM-5 | ST448 |
| 129 | CDE6301 | / | ST405 |
| 130 | CDE6304 | NDM-5 | ST131 |
| 131 | CDE6305 | NDM-7 | ST224 |
| 132 | CDE6306 | NDM-9 | - |
| 133 | CDE6307 | NDM-5 | ST405 |
| 134 | CDE6312 | / | ST209 |
| 135 | CDE6314 | NDM-1 | ST354 |
| 136 | CDE6315 | / | ST46 |
| 137 | CDE6603 | NDM-5 | ST167 |
| 138 | CDE6701 | NDM-5 | ST410 |
| 139 | CDE6702 | NDM-5 | ST1196 |
| 140 | CDE6703 | NDM-5 | ST746 |
| 141 | CDE6801_1 | NDM-5 | - |
| 142 | CDE6801_2 | NDM-5 | - |
| 143 | CDE6902 | / | ST1589 |
| 144 | CDE6904 | NDM-5 | ST410 |
| 145 | CDE7004 | / | ST648 |
| 146 | CDE7101 | NDM-5 | ST354 |
| 147 | CDE7203 | NDM-5 | ST7115 |
| 148 | CDE7403 | NDM-7 | ST746 |
| 149 | CDE7405 | NDM-5 | ST155 |
| 150 | CDE7411 | / | ST457 |
| 151 | CDE7416 | NDM-1 | ST607 |
| 152 | CDE7417 | NDM-1 | ST216 |
| 153 | CDE7418 | NDM-1 | ST457 |
| 154 | CDE7501 | / | ST44 |
| 155 | CDE7502 | NDM-5 | ST167 |
| 156 | CDE7601 | / | ST101 |
| 157 | CDE7603 | / | ST101 |
| 158 | CDE7604 | / | ST101 |
| 159 | CDE7701 | NDM-5 | ST744 |
| 160 | CDE7702 | NDM-5 | ST410 |
| 161 | CDE7703 | NDM-5 | ST361 |
| 162 | CDE7705_1 | NDM-5 | ST10 |
| 163 | CDE7705_2 | NDM-5 | ST2115 |
| 164 | CDE7902 | / | ST4985 |
| 165 | CDE8005 | NDM-5 | ST354 |
| 166 | CDE8008 | NDM-5 | ST648 |
| 167 | CDE8010 | NDM-5 | ST410 |
| 168 | CDE8012 | NDM-5 | ST38 |
| 169 | CDE8013 | NDM-5 | ST648 |
| 170 | CDE8101 | NDM-1 | ST648 |
| 171 | CDE8201 | NDM-5 | ST410 |
| 172 | CDE8202 | NDM-5 | ST349 |
| 173 | CDE8205 | NDM-5 | ST5229 |
| 174 | CDE8207 | / | ST1266 |
| 175 | CDE8301 | NDM-5 | ST156 |
| 176 | CDE8702 | NDM-5 | ST10 |
| 177 | CDE8705 | NDM-5 | ST617 |
| 178 | CDE8706 | NDM-5 | ST405 |
| 179 | CDE8801 | / | - |
| 180 | CDE8901 | NDM-5 | ST10 |
| 181 | CDE9101 | NDM-5 | ST457 |
| 182 | CDE9103 | / | ST354 |
| 183 | CDK8103 | NDM-5 | ST224 |
| 184 | CDS6801 | / | ST101 |

**Table S4 Carbapenemase and STs of intestinal colonized CRKP isolates**

| **No.** | **Isolate_ID** | **Carbapenemase** | **ST** |
| --- | --- | --- | --- |
| 1 | CDE0802 | KPC-2 | ST15 |
| 2 | CDE5101 | KPC-2 | ST11 |
| 3 | CDK0101 | - | ST15 |
| 4 | CDK0103 | - | ST147-1LV |
| 5 | CDK0104 | - | ST147 |
| 6 | CDK0201 | KPC-2 | ST11 |
| 7 | CDK0202 | NDM-5 | ST1699 |
| 8 | CDK0203 | KPC-2;NDM-1 | ST11 |
| 9 | CDK0204_1 | KPC-2 | ST11 |
| 10 | CDK0204_2 | KPC-2 | ST11 |
| 11 | CDK0205 | - | ST3393 |
| 12 | CDK0302 | NDM-5 | ST485 |
| 13 | CDK0304 | - | ST3412 |
| 14 | CDK0401 | KPC-2 | ST11 |
| 15 | CDK0402 | KPC-2 | ST11 |
| 16 | CDK0403 | KPC-2 | ST11 |
| 17 | CDK0404 | KPC-2 | ST15 |
| 18 | CDK0405 | KPC-2 | ST11 |
| 19 | CDK0406 | KPC-2 | ST11 |
| 20 | CDK0407 | KPC-2 | ST3499 |
| 21 | CDK0408 | - | ST3184-1LV |
| 22 | CDK0501 | KPC-2 | ST15 |
| 23 | CDK0502 | - | ST469 |
| 24 | CDK0503 | KPC-2 | ST15 |
| 25 | CDK0504 | KPC-2 | ST11 |
| 26 | CDK0506 | - | ST967 |
| 27 | CDK0507 | - | ST469 |
| 28 | CDK0508 | - | ST281 |
| 29 | CDK0509 | - | ST147 |
| 30 | CDK0510 | - | ST45 |
| 31 | CDK0701 | KPC-2 | ST43 |
| 32 | CDK0702 | KPC-2 | ST412 |
| 33 | CDK0801 | - | ST485 |
| 34 | CDK0802 | - | ST39 |
| 35 | CDK0803 | KPC-2 | ST15 |
| 36 | CDK0804 | - | ST15 |
| 37 | CDK1004 | NDM-4 | ST193 |
| 38 | CDK1006 | - | ST37 |
| 39 | CDK1101 | NDM-5 | ST147 |
| 40 | CDK1102 | KPC-2 | ST15 |
| 41 | CDK1202 | - | ST4514 |
| 42 | CDK1203 | NDM-5 | ST101 |
| 43 | CDK1204 | KPC-2 | ST11 |
| 44 | CDK1205 | KPC-2 | ST11 |
| 45 | CDK1401 | - | ST485 |
| 46 | CDK1402 | - | ST72-3LV |
| 47 | CDK1501 | KPC-2 | ST15 |
| 48 | CDK1601 | KPC-2 | ST15 |
| 49 | CDK1602 | KPC-2 | ST1031 |
| 50 | CDK2001 | - | ST5112 |
| 51 | CDK2201 | OXA-232 | ST15 |
| 52 | CDK2202 | KPC-2 | ST11 |
| 53 | CDK2901 | KPC-2 | ST2388 |
| 54 | CDK2902 | KPC-2 | ST11 |
| 55 | CDK2903 | - | ST15 |
| 56 | CDK3001 | - | ST37 |
| 57 | CDK3301 | NDM-5 | ST485 |
| 58 | CDK3305 | - | ST15 |
| 59 | CDK3306 | - | ST1504-2LV |
| 60 | CDK3402 | - | ST2407 |
| 61 | CDK3403 | NDM-5 | ST15 |
| 62 | CDK3502 | - | ST395 |
| 63 | CDK3601 | NDM-5 | ST485 |
| 64 | CDK3602 | - | ST726-1LV |
| 65 | CDK3603 | - | ST2407 |
| 66 | CDK3701 | KPC-2 | ST11 |
| 67 | CDK4001 | KPC-2 | ST15 |
| 68 | CDK4003 | - | ST229 |
| 69 | CDK4005 | KPC-2 | ST11 |
| 70 | CDK4008 | KPC-2;NDM-1 | ST1378 |
| 71 | CDK4009 | NDM-1;OXA-181 | ST1681 |
| 72 | CDK4102 | KPC-2 | ST11 |
| 73 | CDK4301 | - | ST11-1LV |
| 74 | CDK4302 | - | ST2428 |
| 75 | CDK4401 | NDM-1 | ST3003 |
| 76 | CDK4403 | - | ST25-2LV |
| 77 | CDK4404 | KPC-2 | ST11 |
| 78 | CDK4405 | - | ST25-2LV |
| 79 | CDK4501 | - | ST5556 |
| 80 | CDK4502 | - | ST37 |
| 81 | CDK4702 | KPC-2 | ST11 |
| 82 | CDK4801 | NDM-5 | ST392 |
| 83 | CDK4802 | - | ST485 |
| 84 | CDK4804 | - | ST502 |
| 85 | CDK4901 | NDM-5 | ST1 |
| 86 | CDK4903 | - | ST1128 |
| 87 | CDK4904 | NDM-5 | ST307 |
| 88 | CDK5003 | KPC-2 | ST11 |
| 89 | CDK5004 | - | ST273 |
| 90 | CDK5102 | KPC-2 | ST11 |
| 91 | CDK5103 | KPC-2 | ST11 |
| 92 | CDK5201 | NDM-13 | ST857 |
| 93 | CDK5301 | NDM-5 | ST22 |
| 94 | CDK5302 | - | ST3184-1LV |
| 95 | CDK5401 | KPC-33 | ST11 |
| 96 | CDK5402 | - | ST15 |
| 97 | CDK5404 | - | ST1263 |
| 98 | CDK5405 | KPC-33 | ST11 |
| 99 | CDK5501 | - | ST37 |
| 100 | CDK5502 | KPC-2 | ST15 |
| 101 | CDK5602 | - | ST147 |
| 102 | CDK5701 | - | ST1419 |
| 103 | CDK5803 | - | ST307 |
| 104 | CDK5804 | - | ST2426-1LV |
| 105 | CDK5805 | - | ST562 |
| 106 | CDK5807 | - | ST37 |
| 107 | CDK5808 | - | ST37 |
| 108 | CDK5901 | - | ST534 |
| 109 | CDK6001 | - | ST37 |
| 110 | CDK6102 | KPC-2 | ST11 |
| 111 | CDK6103 | KPC-2 | ST11 |
| 112 | CDK6104 | - | ST273 |
| 113 | CDK6201 | - | ST273 |
| 114 | CDK6202 | - | ST1128 |
| 115 | CDK6303 | - | ST3393 |
| 116 | CDK6304 | - | ST307 |
| 117 | CDK6306 | - | ST3393 |
| 118 | CDK6308 | - | ST967 |
| 119 | CDK6309 | - | ST55 |
| 120 | CDK6310 | - | ST15 |
| 121 | CDK6311 | - | ST15 |
| 122 | CDK6312 | - | ST273 |
| 123 | CDK6313 | - | ST485 |
| 124 | CDK6314 | - | ST307 |
| 125 | CDK6315 | NDM-1 | ST685 |
| 126 | CDK6317 | - | ST485 |
| 127 | CDK6402 | KPC-2 | ST11 |
| 128 | CDK6403 | KPC-2 | ST11 |
| 129 | CDK6404 | - | ST37 |
| 130 | CDK6601 | KPC-2 | ST11 |
| 131 | CDK6803 | - | ST273 |
| 132 | CDK6901 | NDM-5 | ST37 |
| 133 | CDK6903 | - | ST307 |
| 134 | CDK7101 | - | ST262-1LV |
| 135 | CDK7103 | - | ST1920-2LV |
| 136 | CDK7104 | KPC-2 | ST147 |
| 137 | CDK7302 | - | ST101 |
| 138 | CDK7401 | KPC-2 | ST11 |
| 139 | CDK7402 | NDM-5 | ST5288 |
| 140 | CDK7403 | KPC-2 | ST11 |
| 141 | CDK7405 | - | ST828-1LV |
| 142 | CDK7407 | - | ST15 |
| 143 | CDK7503 | - | ST13 |
| 144 | CDK7507 | - | ST15 |
| 145 | CDK7601 | - | ST256 |
| 146 | CDK7701 | NDM-1 | ST622 |
| 147 | CDK7901 | NDM-1 | ST37-1LV |
| 148 | CDK7902 | - | ST36 |
| 149 | CDK8003 | NDM-5 | ST37 |
| 150 | CDK8004 | NDM-5 | ST483 |
| 151 | CDK8102 | - | ST1922 |
| 152 | CDK8202 | - | ST101 |
| 153 | CDK8203 | NDM-5 | ST307 |
| 154 | CDK8701 | KPC-2 | ST11 |
| 155 | CDK8704 | KPC-2 | ST11 |
| 156 | CDK8705 | KPC-2 | ST11 |
| 157 | CDK8706 | KPC-2 | ST11 |
| 158 | CDK8707 | KPC-2 | ST11 |
| 159 | CDK8801 | NDM-1 | ST111 |
| 160 | CDK8802 | IMP-4 | ST3228 |
| 161 | CDK8803 | KPC-2 | ST15 |
| 162 | CDK8804 | KPC-2 | ST15 |
| 163 | CDK8805 | KPC-2 | ST15 |
| 164 | CDK8806 | KPC-2 | ST11 |
| 165 | CDK8807 | KPC-2 | ST15 |
| 166 | CDK8808 | KPC-2 | ST11 |
| 167 | CDK8809 | KPC-2 | ST11 |
| 168 | CDK8902 | NDM-5 | ST11 |
| 169 | CDK8905 | - | ST3393 |
| 170 | CDK9102 | KPC-2 | ST11 |
| 171 | CDK9201 | - | ST36 |

**Table S5 Carbapenemase and STs of intestinal colonized carbapenem-resistant *Enterobacter* spp. isolates**

| **No.** | **Isolate_ID** | **Carbapenemase** | **ST** |
| --- | --- | --- | --- |
| 1 | CDS0201 | NDM-1 | 171 |
| 2 | CDS0203 | NDM-1 | 837 |
| 3 | CDS0401 | / | / |
| 4 | CDS0501 | NDM-1 | 78 |
| 5 | CDS0503 | NDM-1 | 78 |
| 6 | CDS0901 | NDM-1 | 171 |
| 7 | CDS1003 | IMP-4 | 45 |
| 8 | CDS1502 | NDM-1 | 116 |
| 9 | CDS1901 | NDM-1 | 51 |
| 10 | CDS2201 | KPC-2 | 27 |
| 11 | CDS2601 | NDM-1 | 252 |
| 12 | CDS2701 | NDM-1 | 171 |
| 13 | CDS4701 | NDM-5 | 177 |
| 14 | CDS5301 | NDM-5 | 171 |
| 15 | CDS5302 | NDM-1 | 114 |
| 16 | CDS5303 | NDM-1 | 114 |
| 17 | CDS5304 | NDM-5 | 1120 |
| 18 | CDS5501 | NDM-1 | 171 |
| 19 | CDS5802 | NDM-5 | 1120 |
| 20 | CDS6104 | / | 702 |
| 21 | CDS6105 | NDM-1 | / |
| 22 | CDS6106 | NDM-1 | 92 |
| 23 | CDS6303 | NDM-1 | 66 |
| 24 | CDS6304 | NDM-1 | 78 |
| 25 | CDS6402 | NDM-1 | 190 |
| 26 | CDS7102 | NDM-5 | 418 |
| 27 | CDS7407 | NDM-1 | / |
| 28 | CDS7409 | NDM-1 | / |
| 29 | CDS7501 | KPC-2 | 78 |
| 30 | CDS7901 | IMP-26 | 175 |
| 31 | CDS7902 | NDM-1 | 270 |
| 32 | CDS7903 | NDM-1 | 1073 |
| 33 | CDS8101 | / | 420 |
| 34 | CDS8201 | NDM-1 | 78 |
| 35 | CDS8701 | / | / |
| 36 | CDS8702 | NDM-1 | 45 |
| 37 | CDS8801 | NDM-1 | 78 |
| 38 | CDS8803 | NDM-1 | 78 |
| 39 | CDS8804 | NDM-1 | 78 |
| 40 | CDS8805 | NDM-1 | 78 |
| 41 | CDS8902 | / | 414 |
| 42 | CDS9201 | / | 133 |

**Table S6 Carbapenemase and STs of intestinal colonized carbapenem-resistant *K. oxytoca* isolates**

| **No.** | **Isolate_ID** | **Carbapenemase** | **ST** |
| --- | --- | --- | --- |
| 1 | CDS0202 | NDM-1 | 29 |
| 2 | CDS0204 | IMP-4 | / |
| 3 | CDS0301 | NDM-1 | 145 |
| 4 | CDS0801 | KPC-2 | 86 |
| 5 | CDS1701 | NDM-1 | 330 |
| 6 | CDS2301 | NDM-1 | 29 |
| 7 | CDS8301 | / | 22 |
| 8 | CDS8402 | NDM-1 | 43 |
| 9 | CDS8806 | NDM-1、IMP-4 | 330 |

**Table S7 Carbapenemase and STs of intestinal colonized carbapenem-resistant *K. aerogenes* isolates**

| **No.** | **Isolate_ID** | **Carbapenemase** | **ST** |
| --- | --- | --- | --- |
| 1 | CDS0702 | OXA-181 | 240 |
| 2 | CDS0703 | OXA-181 | 240 |
| 3 | CDS1001 | OXA-181 | 261 |
| 4 | CDS1702 | NDM-5 | 267 |
| 5 | CDS3001 | / | 14 |
| 6 | CDS7101 | / | 267 |
| 7 | CDS7404 | / | 600 |

**Table S8 Carbapenemase and STs of intestinal colonized carbapenem-resistant CRPA isolates**

| **No.** | **Isolate_ID** | **Carbapenemase** | **ST** |
| --- | --- | --- | --- |
| 1 | CDP0101 | / | 16 |
| 2 | CDP0201 | / | 1239 |
| 3 | CDP0202 | KPC-2 | 463 |
| 4 | CDP0203 | KPC-2 | 463 |
| 5 | CDP0301 | / | 3217 |
| 6 | CDP0402 | AFM-1、KPC-2 | 463 |
| 7 | CDP0501 | / | 179 |
| 8 | CDP0601 | / | 252 |
| 9 | CDP0801 | KPC-2 | 463 |
| 10 | CDP0802 | / | 244 |
| 11 | CDP1002 | / | 389 |
| 12 | CDP1101 | / | 270 |
| 13 | CDP1202 | / | 782 |
| 14 | CDP1204 | / | 260 |
| 15 | CDP1303 | / | 1475 |
| 16 | CDP1501 | / | 487 |
| 17 | CDP1502 | / | 532 |
| 18 | CDP1601 | / | 611 |
| 19 | CDP1603 | / | 508 |
| 20 | CDP1604 | / | 385 |
| 21 | CDP1701 | / | 1567 |
| 22 | CDP1703 | / | 408 |
| 23 | CDP1704 | / | 274 |
| 24 | CDP2001 | KPC-2 | 463 |
| 25 | CDP2101 | / | 360 |
| 26 | CDP2301 | / | 242 |
| 27 | CDP2601 | / | 471 |
| 28 | CDP2602 | / | 1971 |
| 29 | CDP2901 | / | 2726 |
| 30 | CDP2902 | / | 316 |
| 31 | CDP2903 | / | 1393 |
| 32 | CDP2905 | / | 303 |
| 33 | CDP3301 | / | 244 |
| 34 | CDP3302 | / | 446 |
| 35 | CDP3501 | / | 162 |
| 36 | CDP3601 | / | 606 |
| 37 | CDP3604 | / | 1052 |
| 38 | CDP3606 | / | 623 |
| 39 | CDP3607 | / | 1437 |
| 40 | CDP3608 | / | 3959 |
| 41 | CDP3609 | / | 273 |
| 42 | CDP3610 | / | 1693 |
| 43 | CDP3701 | / | 274 |
| 44 | CDP3702 | / | 550 |
| 45 | CDP3703 | / | 360 |
| 46 | CDP3801 | / | 644 |
| 47 | CDP4001 | / | 319 |
| 48 | CDP4101 | / | 2629 |
| 49 | CDP4102 | / | 970 |
| 50 | CDP4103 | / | 357 |
| 51 | CDP4201 | / | 773 |
| 52 | CDP4401 | / | 1337 |
| 53 | CDP4402 | / | 313 |
| 54 | CDP4501 | / | 792 |
| 55 | CDP4701 | / | 856 |
| 56 | CDP4801 | / | 3393 |
| 57 | CDP4901 | / | 245 |
| 58 | CDP4902 | / | 260 |
| 59 | CDP5001 | / | 274 |
| 60 | CDP5101 | / | 480 |
| 61 | CDP5201 | / | 233 |
| 62 | CDP5301 | / | 1453 |
| 63 | CDP5302 | / | 111 |
| 64 | CDP5401 | / | 671 |
| 65 | CDP5402 | / | 357 |
| 66 | CDP5403 | / | 144 |
| 67 | CDP5404 | / | 277 |
| 68 | CDP5501 | / | 244 |
| 69 | CDP5701 | / | 260 |
| 70 | CDP5801 | / | 27 |
| 71 | CDP5802 | / | - |
| 72 | CDP5803 | / | 683 |
| 73 | CDP5805 | / | 260 |
| 74 | CDP5808 | / | 856 |
| 75 | CDP5809 | / | 557 |
| 76 | CDP5903 | / | 1020 |
| 77 | CDP6002 | / | 3134 |
| 78 | CDP6201 | / | 260 |
| 79 | CDP6202 | / | 1437 |
| 80 | CDP6301 | / | 683 |
| 81 | CDP6302 | / | 3959 |
| 82 | CDP6303 | / | 281 |
| 83 | CDP6304 | / | 412 |
| 84 | CDP6305 | / | 313 |
| 85 | CDP6307 | / | 798 |
| 86 | CDP6402 | / | 277 |
| 87 | CDP6601 | / | 277 |
| 88 | CDP6602 | / | 244 |
| 89 | CDP6603 | / | 252 |
| 90 | CDP6604 | / | 508 |
| 91 | CDP6701 | / | 244 |
| 92 | CDP6702 | / | 1684 |
| 93 | CDP6904 | / | 209 |
| 94 | CDP7001 | / | 1428 |
| 95 | CDP7002 | / | 270 |
| 96 | CDP7102 | / | 827 |
| 97 | CDP7201 | / | - |
| 98 | CDP7302 | VIM-2 | 277 |
| 99 | CDP7401 | / | 549 |
| 100 | CDP7402 | / | 3337 |
| 101 | CDP7403 | / | 267 |
| 102 | CDP7405 | / | 553 |
| 103 | CDP7407 | / | 2235 |
| 104 | CDP7408 | / | 1455 |
| 105 | CDP8001 | / | 357 |
| 106 | CDP8007 | / | 9 |
| 107 | CDP8101 | / | 1094 |
| 108 | CDP8201 | / | 485 |
| 109 | CDP8202 | / | 379 |
| 110 | CDP8203 | / | 1212 |
| 111 | CDP8304 | / | 1455 |
| 112 | CDP8402 | / | 406 |
| 113 | CDP8701 | / | 3083 |
| 114 | CDP8801 | / | - |
| 115 | CDP8901 | / | 357 |
| 116 | CDP8902 | / | 1756 |
| 117 | CDP9001 | / | 645 |
| 118 | CDP9102 | / | 494 |
| 119 | CDP9103 | / | 553 |
| 120 | CDP9104 | / | 4267 |
| 121 | CDP9105 | / | 1663 |

**Table S9 Carbapenemase and STs of intestinal colonized carbapenem-resistant CRAB isolates**

| **No.** | **Isolate_ID** | **Carbapenemase** | | **ST（Pas）** | **ST（Oxford）** |
| --- | --- | --- | --- | --- | --- |
| 1 | CDA0601 | OXA-66 | OXA-23 | 2 | 1806/208 |
| 2 | CDA1201 | OXA-72 | OXA-429 | / | 1708 |
| 3 | CDA3001 | OXA-66 | OXA-23 | 2 | 1837/369 |
| 4 | CDA3702 | OXA-66 | OXA-23 | 2 | 1837/369 |
| 5 | CDA4002 | OXA-23 | OXA-64 | 25 | 229 |
| 6 | CDA4401 | NDM-1 | OXA-58 | / | 2078 |
| 7 | CDA4402 | NDM-1 | OXA-72 | / | 2078 |
| 8 | CDA5101 | OXA-72 |  | 651 | / |
| 9 | CDA5301 | OXA-510 | OXA-58 | 1223 | 2449 |
| 10 | CDA5701 | OXA-66 | OXA-23 | 2 | 1806/208 |
| 11 | CDA6401 | OXA-66 | OXA-23 | 2 | 1806/208 |
| 12 | CDA6402 | OXA-66 | OXA-23 | 2 | 540 |
| 13 | CDA7401 | OXA-66 | OXA-23 | 2 | 1806/208 |
| 14 | CDA8002 | OXA-66 | OXA-23 | 2 | 1806/208 |
| 15 | CDA8301 | OXA-66 | OXA-23 | 2 | 1816/195 |

**Table S10 The swabs collection date of each hospital in the study.**

| No. | Hospital | Collection date |
| --- | --- | --- |
| 01 | Jinhua municipal central hospital Medical group | 2021/7/20 |
| 02 | Zhejiang Provincial Hospital of Chinese Medicine | 2021/7/23 |
| 03 | Zhejiang Provincial People‘s hospital | 2021/8/3 |
| 04 | Tongde hospital of Zhejiang Province | 2021/8/3 |
| 05 | Affiliated Hangzhou first people's hospital | 2021/8/3 |
| 06 | The second affiliated hospital Zhejiang university school of medicine | 2021/8/5 |
| 07 | Sir Run Run Shaw Hospital Zhejiang University school of medicine | 2021/8/5 |
| 08 | The first affiliated hospital Zhejiang university school of medicine | 2021/8/5 |
| 09 | Huzhou central hospital | 2021/8/9 |
| 10 | The affiliated people's hospital of Ningbo university | 2021/8/10 |
| 11 | Taizhou central hospital | 2021/8/10 |
| 12 | The first affiliated hospital of Ningbo university | 2021/8/10 |
| 13 | Ningbo No.2 hospital | 2021/8/11 |
| 14 | Jinhua people's hospital | 2021/8/11 |
| 15 | Taizhou hospital of Zhejiang province | 2021/8/12 |
| 16 | The first affiliated hospital of Wenzhou medical university | 2021/8/13 |
| 17 | Shaoxing people's hospital | 2021/8/13 |
| 18 | The first hospital of Jiaxing | 2021/8/13 |
| 19 | Quzhou people's hospital | 2021/8/17 |
| 20 | The affiliated hospital of Hangzhou normal university | 2021/8/17 |
| 21 | Children's hospital of Zhejiang university school of medicine | 2021/8/17 |
| 22 | Zhejiang hospital | 2021/8/17 |
| 23 | Dongyang people's hospital | 2021/8/18 |
| 24 | The first people’s hospital of Yuhang District | 2021/8/18 |
| 25 | Wenzhou central hospital | 2021/8/19 |
| 26 | Lishui central hospital | 2021/8/19 |
| 27 | Yiwu central hospital | 2021/8/19 |
| 28 | Zhejiang Quhua hospital | 2021/8/20 |
| 29 | Shanghai General Hospital | 2021/8/31 |
| 30 | Rujin hospital Shanghai jiaotong university of medicine | 2021/8/31 |
| 31 | Qilu hospital of Shandong university (Qingdao) | 2021/8/31 |
| 32 | Anhui provincial hospital | 2021/8/31 |
| 33 | Qilu hospital of Shandong university | 2021/8/31 |
| 34 | Children's Hospital of Fudan University | 2021/9/1 |
| 35 | Jiujiang No.1 people's hospital | 2021/9/1 |
| 36 | The second affiliated hospital of Harbin medical university | 2021/9/1 |
| 37 | Affiliated hospital of Nantong University | 2021/9/8 |
| 38 | Fujian provincial hospital | 2021/9/8 |
| 39 | Henan cancer hospital | 2021/9/8 |
| 40 | The first affiliated hospital of Zhengzhou university | 2021/9/8 |
| 41 | The First Affiliated Hospital of Fujian medical university | 2021/9/8 |
| 42 | The first affiliated hospital of Sun Yat-sen University | 2021/9/10 |
| 43 | Guangzhou first people's hospital | 2021/9/10 |
| 44 | Nanfang Hospital, Southern Medical University | 2021/9/10 |
| 45 | Zhongshan hospital Xiamen university | 2021/9/13 |
| 46 | Shenzhen children's hospital | 2021/9/13 |
| 47 | Henan Provincial People‘s hospital | 2021/9/13 |
| 48 | The second people's hospital of Shenzhen | 2021/9/14 |
| 49 | The first affiliated hospital of Xi'an Jiaotong university | 2021/9/15 |
| 50 | Fujian medical university union hospital | 2021/9/15 |
| 51 | Zhujiang hospital of Southern medical university | 2021/9/15 |
| 52 | Xiangyang central hospital | 2021/9/15 |
| 53 | The first hospital of Jilin University | 2021/9/15 |
| 54 | Xiangya hospital Central South University | 2021/9/15 |
| 55 | The first hospital of China medical university | 2021/9/15 |
| 56 | The first people’s hospital of Yunnan Province | 2021/9/15 |
| 57 | The People's Liberation Army General Hospital of Chengdu Command | 2021/9/15 |
| 58 | Institute of hematology & blood diseases hospital, Chinese academy of medical science & Peking union medical college | 2021/9/15 |
| 59 | The first affiliated hospital of Guangxi medical university | 2021/9/15 |
| 60 | Xijing hospital of AFMU | 2021/9/15 |
| 61 | The first affiliated hospital of Soochow university | 2021/9/23 |
| 62 | Shaanxi province people's hospital | 2021/9/23 |
| 63 | Peking university people's hospital | 2021/9/27 |
| 64 | The Second Affiliated Hospital of AFMU | 2021/9/27 |
| 65 | The second hospital of Hebei medical university | 2021/9/27 |
| 66 | Chinese PLA General Hospital | 2021/10/9 |
| 67 | Hainan general hospital | 2021/10/9 |
| 68 | Xinqiao Hospital, Army Medical University | 2021/10/9 |
| 69 | Hebei Yanda Lu Daopei hospital | 2021/10/9 |
| 70 | The affiliated hospital of Inner Mongolia medical university | 2021/10/19 |
| 71 | The second hospital of Dalian medical university | 2021/10/19 |
| 72 | The 920 Hospital of the Joint Service Support Force of the People's Liberation Army of China | 2021/10/25 |
| 73 | First affiliated hospital of Kunming medical university | 2021/10/25 |
| 74 | The first affiliated hospital of Nangchang university | 2021/10/25 |
| 75 | The first affiliated hospital of Harbin medical university | 2021/10/28 |
| 76 | Shandong Provincial Hospital Affiliated to Shandong First Medical University | 2021/10/28 |
| 77 | The first affiliated hospital of Anhui medical university | 2021/10/29 |
| 78 | Tibet People's hospital | 2021/10/30 |
| 79 | Shanxi Bethune Hospital, Shanxi Academy of Medical Sciences | 2021/11/19 |
| 80 | The first affiliated hospital of Guizhou medical university | 2021/11/23 |
| 81 | The first affiliated hospital of Chongqing medical university | 2021/11/23 |
| 82 | Tongji Hospital affiliated to Tongji Medical College of Huazhong University of Science & Technology | 2021/11/24 |
| 83 | General hospital of Ningxia medical university | 2021/11/24 |
| 84 | The First hospital of Shanxi medical university | 2021/11/24 |
| 85 | Qinghai university affiliated hospital | 2021/11/24 |
| 86 | Gansu province hospital | 2021/11/24 |
| 87 | West China Hospital of Sichuan University | 2021/11/27 |
| 88 | Tongji Hospital of Tongji University | 2021/11/29 |
| 89 | Tianjin medical university general hospital | 2021/12/7 |
| 90 | Lanzhou university second hospital | 2021/12/7 |
| 91 | The first affiliated hospital of Xinjiang medical university | 2021/12/8 |
| 92 | Xi'an international medical center hospital | 2021/12/8 |
